# Significant Improvement of Mechanical Properties of SiC-Nanowire-Reinforced SiC_f_/SiC Composites via Atomic Deposition of Ni Catalysts

**DOI:** 10.3390/ma15082900

**Published:** 2022-04-15

**Authors:** Zongxu Wu, Haoran Wang, Zhaoke Chen, Ruiqian Zhang, Qingbo Wen, Zongbei He, Ming Li, Xiang Xiong

**Affiliations:** 1 Science and Technology on High Strength Structural Materials Laboratory, Central South University, Changsha 410083, China; mischief_joean@outlook.com (Z.W.); haoran414@163.com (H.W.); wentsingbo@csu.edu.cn (Q.W.); 2 State Key Laboratory of Powder Metallurgy, Central South University, Changsha 410083, China; xiongx@csu.edu.cn; 3 Science and Technology on Reactor Fuel and Materials Laboratory, Nuclear Power Institute of China, Chengdu 610213, China; zhang_ruiqian@126.com (R.Z.); hezongbei@126.com (Z.H.); ming_li_npic@163.com (M.L.)

**Keywords:** SiC nanowires, atomic deposition method, SiC_f_/SiC composites, compression performance

## Abstract

This study aimed to study the effects of different catalyst introduction methods on the distribution of SiC nanowires (SiCNWs) and the mechanical properties of SiC_f_/SiC composites. Two different catalyst-introduction methods (electroplating (EP) vs. atomic deposition (AD)) have been used to catalyze the growth of SiC nanowires in SiC_f_ preforms. The morphology, structure and phase composition were systematically investigated using scanning electron microscopy (SEM), transmission electron microscopy (TEM) and X-ray diffraction (XRD). The SiCNWs-reinforced SiC_f_/SiC composited was densified by CVI. The compressive strength of the SiCNWs-reinforced SiC_f_/SiC composites was evaluated by radial crushing test. Compared with EP, atomic Ni catalysts fabricated by AD have higher diffusivity for better diffusion into the SiC_f_ preform. The yield of SiCNWs is effectively increased in the internal pores of the SiC_f_ preform, and a denser network forms. Therefore, the mechanical properties of SiCNW-containing SiC_f_/SiC composites are significantly improved. Compared with the EP-composites and SiC_f_/SiC composites, the compressive strength of AD-composites is increased by 51.1% and 56.0%, respectively. The results demonstrate that the use of AD method to grow SiCNWs is promising for enhancing the mechanical properties of SiC_f_/SiC composites.

## 1. Introduction

Silicon-carbide-fiber-reinforced silicon carbide matrix (SiC_f_/SiC) composites are characterised by high specific strength, high specific modulus [1], high temperature resistance [2], and good radiation resistance properties [3]. The unique combination of these desirable properties makes SiC_f_/SiC composites cladding tube are deemed the ideal accident tolerant fuel (ATF) cladding materials [4,5,6,7]. However, due to the accumulation of helium and fission gas in the tube, additional stress would be generated by internal pressure during service in a nuclear reactor, leading the material to breakdown [8,9]. Therefore, it is necessary to improve the mechanical properties of the composites to enable the safe operation of nuclear reactors.

In order to improve the mechanical properties of SiCf/SiC cladding tube, in our previous study [10], we demonstrated that the 2.5D shallow bend-joint preform structure can achieve the highest compressive strength after full densification. However, since the micron SiC fiber cannot resist the microcracks in the matrix [11], SiC fiber is difficult to be used as an effective reinforcing phase in the submicron scale of the matrix. Therefore, nanoscale materials should be introduced into SiC matrix as the second reinforcements. Among the nanoscale materials, one-dimensional materials are considered to be suitable as nano-fibers to reinforce micro-domains of composites due to their excellent elasticity, flexibility, and high tensile strength [12]. SiC nanowires (SiCNWs), usually formed as β-SiC phase [13], are most suitable for the reinforcement phase of SiC/SiC cladding tubes in a nuclear environment. Up to date, many efforts have been devoted to introducing SiC nanowires into SiC_f_/SiC composites. Tao et al. [14] fabricated dense three-layer SiC tubes by introducing SiCNWs on the innermost layer, which resulted in SiC cladding tube exhibited an average hoop strength of 316.6 MPa. Kang et al. [15] introduced SiCNWs on the SiC fiber with pyrolytic carbon (PyC) coating via CVI process; and the tensile strength of SiCNWs containing SiC_f_/SiC mini-composites was increased by more than 8%. Han et al. [1] successfully improved the mechanical properties and high-temperature microwave absorption properties of the composites by introducing nanowires into the bulk SiC_f_/SiC composites. Cui et al. [16] successfully synthesized 3D SiC/SiC composites modified by CVI SiC nanowires. The flexural strength of the composites was found to increase by 46%, and the thermal conductivity showed an obvious increase at 25–1000 °C. However, in the studies mentioned above, few studied concerned on the effect of the distribution of SiCNWs on the mechanical propreties of the composites.

The distribution of SiCNWs in the SiC_f_ preforms can be controlled by adjusting the distribution of catalyst. However, catalyst particles could not be easily introduced into the central part of the preforms by traditional catalyst introducing methods, such as, electroplating (EP) [17] and magnetron-sputtering [18]; instead, the catalyst tends to aggregate on the surface of the preforms and blocking the reactant gas diffusing into the inside pores of the preforms [19,20]. To solve this problem, an atomic deposition (AD) method was used in this work to change the distribution of catalyst in SiC_f_ preforms. In the AD process, the catalyst can diffuse into the small pores and be absorbed on the surface of the wall of pores in SiCf preforms easily, avoiding the aggregation of catalyst particles on the surface layer of the preform. The distribution of SiC nanowires can be regulated by AD method, which is expected to improve the mechanical properties of SiC_f_/SiC composites greatly.

In the present work, in-situ grown SiC nanowires were introduced into SiCf preforms by two different catalyst introduction method (EP and AD). The influence of different catalyst introduction methods on the morphology and distribution of the SiCNWs was characterised in detail by scanning electron microscopy (SEM) and transmission electron microscopy (TEM). In addition, the effects of EP and AD methods on the density, microstructure and mechanical properties of the composites were studied.

## 2. Materials and Methods

### 2.1. Materials

Nickel sulphate hexahydrate (NiSO_4_·6H_2_O), nickel chloride (Ni_2_Cl), boric acid (H_3_BO_3_), and sodium dodecyl sulphate (C_12_H_25_SO_4_Na) of analytical reagent (AR) grade were purchased from Sigma-Aldrich Co., LLC (St. Louis, MI, USA). Cansas-III SiC fiber bundles and methyl trichlorosilane (MTS; 99% purity) were obtained from Leaoasia New Material Co., Ltd. (Quanzhou, China) and Luxi Chemical Group Co., Ltd. (Liaocheng, China), respectively. H_2_ and Ar gas with a purity of 99.99% were purchased from Changsha Hi-Tech Gas Co., Ltd. (Changsha, China).

### 2.2. Preparation of Ring Samples

Cansas-III SiC fiber bundles with 500 monofilaments were used to fabricate SiC_f_ cladding tube preforms with an outer diameter of 12 mm, inner diameter of 10 mm, and wall thickness of 1 mm. PyC with a thickness of below 1 μm was introduced on the SiC fiber surface in the preforms as a fiber/matrix interphase. A diamond wire saw was used to cut the cladding tube preforms into ring-shaped specimens with a height of 10 mm. The ring samples were then cleaned and dried for subsequent growth of nanowires.

### 2.3. Electroplating (EP) Ni-Catalyst-Assisted Growth of SiCNWs

The cleaned and dried preforms were used as substrates for the growth of SiCNWs. First, the EP solution was obtained via mixing NiSO_4_·6H_2_O (260 g/L), NiCl_2_ (40 g/L), H_3_BO_3_ (40 g/L), and C_12_H_25_SO_4_Na (0.2 g/L) in 1 L of deionised water by magnetic stirring. After stirring the solution for 1 h, a graphite sheet and the ring-shaped preforms were simultaneously immersed into the solution, and served as the anode and cathode, respectively. Subsequently, an electric current was applied using a DC power supply. Different EP currents (1, 1.5, and 2 A) were used to vary the amount of catalyst, and the corresponding samples were denoted as E-1, E-1.5, and E-2, respectively. The entire EP process was carried out at 25 °C [21] with an EP time of 2 min. The specimens were washed in absolute ethanol by ultrasonication and dried at 50 °C for 24 h. The Ni-plated specimens were then suspended in a CVI furnace for SiCNW growth. MTS was used as the Si and C source and H_2_ was used as the carrier and reaction gas. The growth process was carried out at 1000–1100 °C and 2 kPa for 2 h, with a carrier gas flow rate of 50 sccm and dilute gas flow rate of 750 sccm.

### 2.4. Atomic Deposition (AD) Ni-Catalyst-Assisted Growth of SiCNWs

The Ni catalyst was introduced on the surface of a graphite sheet instead of the SiC_f_ preforms via EP. The EP process was performed in a mixture of NiSO_4_·6H_2_O (16.25 g/L), NiCl_2_ (2.5 g/L), H_3_BO_3_ (2.5 g/L), and C_12_H_25_SO_4_Na (1.25 × 10^−2^ g/L) in 1 L of deionised water, and the current and EP time were 1 A and 2 min, respectively. Subsequently, both the Ni-plated graphite sheet and unplated SiC_f_ preform were suspended in a CVI furnace, which is different from the procedure followed in the EP method. The growth process was carried out at 1000–1100 °C and 2 kPa for 1, 3, and 5 h at a carrier gas flow rate of 50 sccm and dilute gas flow rate of 750 sccm. The corresponding samples were denoted as AD-1, AD-3, and AD-5, respectively. During the heating process, the Ni coating on the graphite sheet forms Ni atoms, which diffuse into the ring specimen and then catalyse the growth of SiCNWs.

### 2.5. Preparation of SiCNW-Containing SiCf/SiC Cladding Tubes

The as-prepared SiCNW-containing SiC_f_ preforms were subjected to a CVI process for densification. The densification process was carried out at 1000–1100 °C and 400–600 Pa for 100 h in a 450-sccm carrier gas and 300-sccm Ar mixed atmosphere.

**Figure 1:**
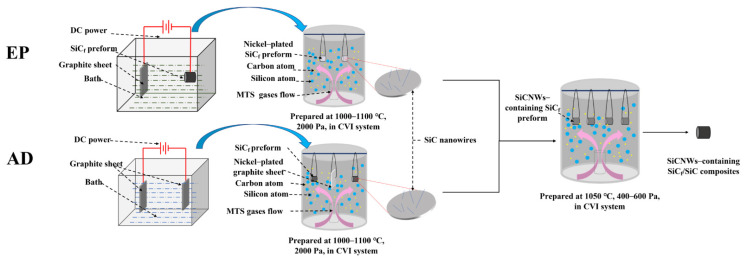
Preparation routes of SiCNW-containing SiC_f_/SiC composites.

The preparation routes of SiCNW-containing SiC_f_/SiC composites are shown in Figure 1. The relevant process parameters for the in-situ growth of SiCNWs and CVI process are shown in Table 1.

### 2.6. Microstructure and Property Characterisation

The density and open porosity of SiCNW-containing SiC_f_/SiC composites were measured using Archimedes’ method. The specimens were weighed using an analytical balance to measure the dry weight (*m*_1_), floating weight (*m*_2_), and saturated weight (*m*_3_). Taking the high open porosity of the SiC_f_/SiC composite material into consideration, the density (*ρ*) and open porosity (*ε*) of the specimens were calculated from the following formulas:

(1) $$\rho =\frac{m_{1}\cdot \rho_{L}}{m_{3}-m_{2}}$$

(2) $$\varepsilon =\frac{m_{3}-m_{1}}{m_{3}-m_{2}}\times 100\%$$

where *ρ_L_* is the density of alcohol (0.789 g/cm^3^).

Radial crushing was performed using an electronic universal testing machine (Instron 3369, Instron Ltd., Norwood, MA, USA). The sample was vertically placed between two benches in the tester. The machine was operated in displacement-control mode and a constant displacement rate of 1.00 mm/min was maintained until the ring sample broke. The maximum compressive load and corresponding compressive displacement of the sample were recorded.

The compressive strength (*σ*) of the specimen was calculated using Equation (3).

(3) $$\sigma =\frac{F\left(D-\delta \right)}{L\delta^{2}}$$

where *F* is the compression load (N), and *D* is the outer diameter (mm), *L* is the length (mm), and *δ* is the wall thickness (mm).

The morphology and distribution of the SiCNWs on the surface and in the cross-sections of the preforms and the fracture morphology of the samples after mechanical testing was observed using a desktop field-emission scanning electron microscope (ProX, Phenom Ltd., Amstelveen, The Netherlands) and field-emission scanning electron microscope (Quanta FEG 250, FEI Ltd., Brno-Černovice, Czech Republic). Low-magnification TEM and high-resolution TEM (HRTEM) images of the SiCNWs were obtained using a JEM-2100F instrument (JEOL Ltd., Tokyo, Japan).

## 3. Results and Discussion

### 3.1. Morphology of the Synthesised SiCNWs

**Figure 2:**
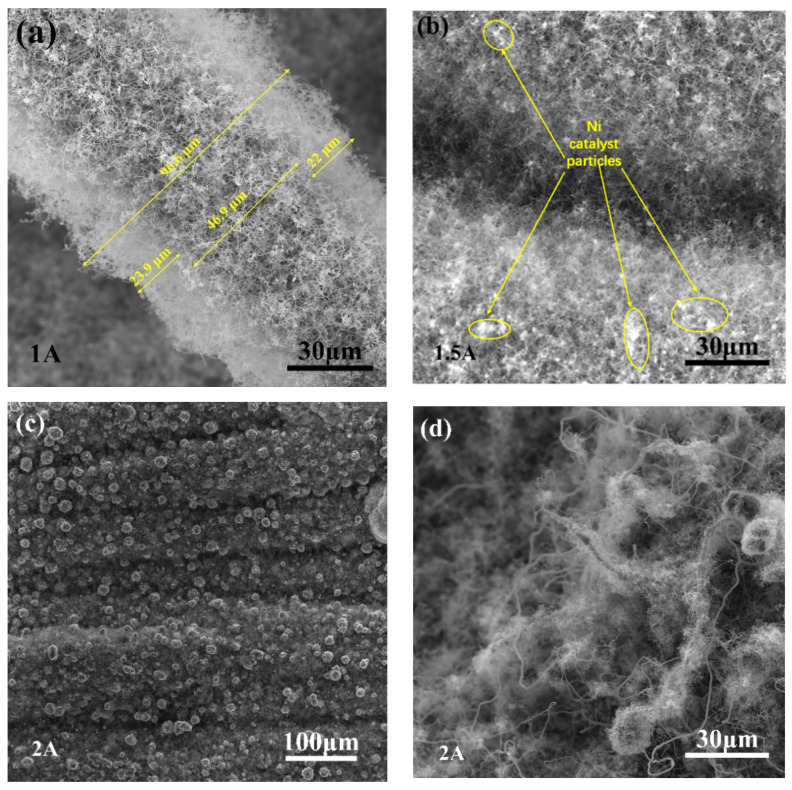
Microstructures of SiCNWs grown via EP with electroplating currents of (**a**) 1 A, (**b**) 1.5 A, and (**c**,**d**) 2 A.

The morphologies of the SiCNWs on the SiC_f_ tube preforms at different EP currents are presented in Figure 2. At a current of 1 A, randomly oriented curved SiCNWs that were tens of micrometres in length with a large aspect ratio were deposited on the SiC fibers (Figure 2a). The growth of SiCNWs along random orientations was attributed to several factors, such as the weak airflow impact force, other attractive/repulsive forces, or driving forces due to a slight temperature or pressure difference [1,22]. At a current of 1.5 A, the SiCNWs were more densely packed. The catalyst particles were observed at the tip of the SiCNWs (Figure 2b), indicating a vapour–liquid–solid (VLS) growth mechanism [23]. With a further increase in current to 2 A, more SiCNWs were obtained. The SiCNW layer on the preform surface was thicker and denser than those produced at lower currents (Figure 2c,d). According to the VLS mechanism, the formed SiC crystal nuclei preferentially precipitate at the interface between the catalyst droplet and fiber surface. A dense continuous Ni catalyst layer (Figure 2c) makes the diffusion of carbon and silicon atoms into the fiber surface challenging.

**Figure 3:**
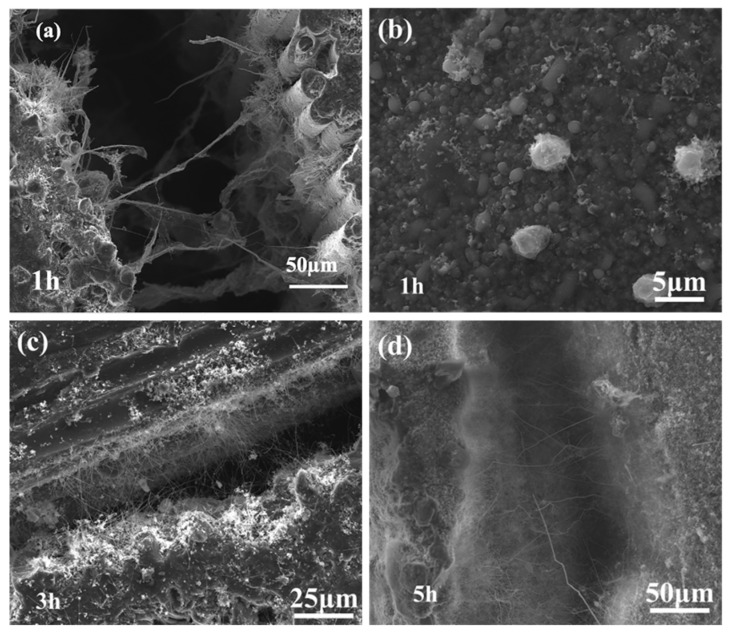
Microstructure of SiCNWs grown via AD method with reaction times of (**a**,**b**) 1 h, (**c**) 3 h, and (**d**) 5 h.

**Figure 4:**
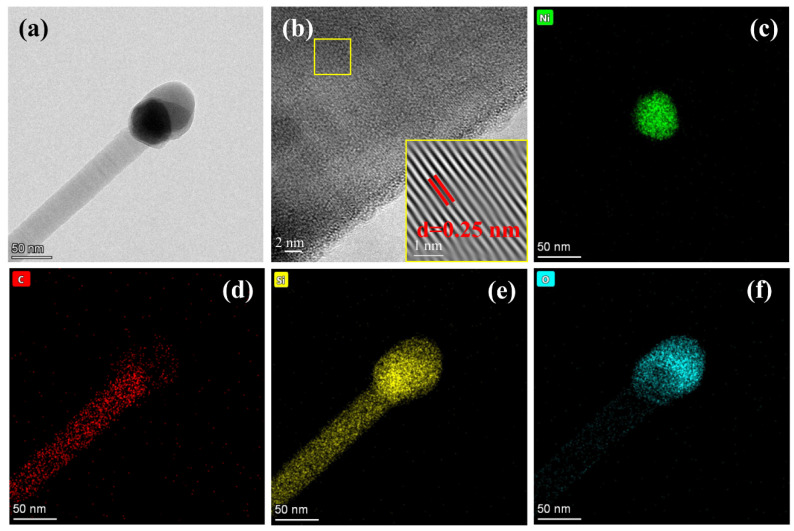
(**a**) TEM image of AD-3 sample, (**b**) HRTEM image of SiCNWs, (**c**–**f**) elemental mapping of sample AD-3.

The morphology of SiCNWs grown using the AD method is shown in Figure 3. A relatively short reaction time disrupted the supply of MTS and the formation of gas-phase Si and C; a short reaction time of 1 h was not conducive to the growth of SiCNWs (Figure 3a,b). With the extension of the deposition time to 3 h, longer SiCNWs were distributed uniformly, and no obvious SiCNW agglomeration was observed between the inter-SiC fiber bundles (Figure 3c); this showed that the SiCNWs were connected but did not block the pores. As shown in Figure 4a, Ni catalyst is placed on the top of SiC nanowires, indicating that the SiCNWs synthesized by AD method conform to V-L-S growth characteristics. The HRTEM image (Figure 4b) shows that the crystal lattice spacing of the SiCNWs was 0.25 nm, which is in accordance with the (111) crystal planes of β-SiC; this indicated that the SiCNWs grew along the (111) direction [24,25]. In addition, an irregular amorphous SiO_2_ layer was observed on the SiCNW surface, which has been attributed to the presence of a small amount of oxygen in the protection atmosphere [1]. The elemental mapping of AD-3 shows that the particle on the top of SiCNWs are composed of Si, O, C and Ni. C, Si are uniformly distributed in SiC nanowires (Figure 4c–f). When the deposition time was increased to 5 h, both the length and number of SiCNWs increased. As shown in Figure 3d, the length of the SiCNWs reached hundreds of micrometres, and the number of nanowires inside the fiber bundle increased significantly.

**Figure 5:**
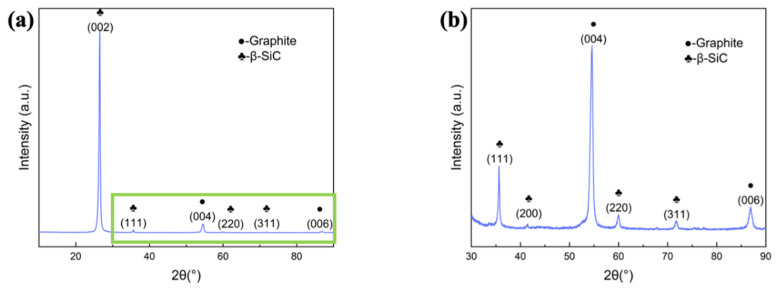
(**a**) The XRD pattern of SiCNWs prepared by AD-3 on graphite sheet, (**b**) magnified patterns of the selected area in (**a**).

To avoid the interference of substrate SiC fibers, SiCNWs were prepared on graphite sheets by AD-3 process, with the results shown in Figure 5. Figure 5a and the enlarged Figure 5b show the main peak at 26°, corresponding to (002) plane of graphite; and some other peaks at 35.5°, 41.3°, 60.3°, and 71.6°, which corresponding to (111), (200), (220), and (311) of β-SiC. The results verified that the one-dimensional nanostructure grown by AD method is β-SiC nanowires.

**Figure 6:**
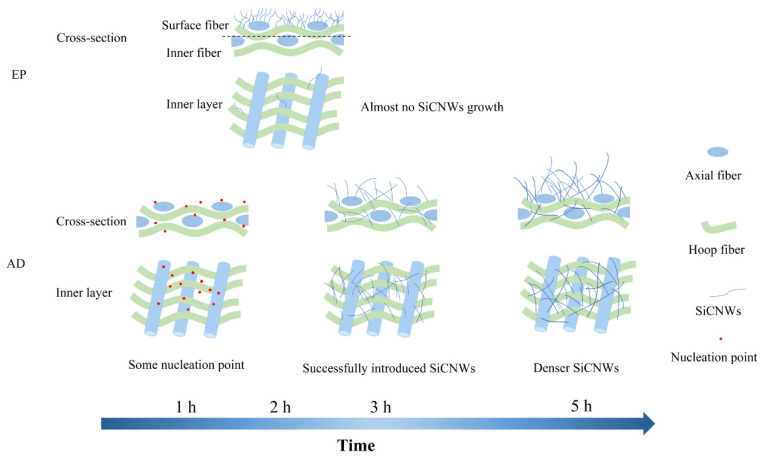
Schematic diagram of the growth mechanism of SiCNWs by EP and AD methods.

Based on the results and discussion mentioned above, a schematic diagram (Figure 6) is proposed to show the advantages of the AD method. When using this method, the Ni catalyst can diffuse into the SiC_f_ preform in the form of gaseous atoms. Therefore, the Ni catalyst introduced by the AD method is more uniform than that introduced by EP. Moreover, the Ni catalysts can be deposited both in the inter-bundle pores and in the inter-laminar layers of the samples. As a result, the SiCNWs are able to grow in the pores. Benefiting from this behaviour, macropores in the preforms can be divided into many small micropores by these SiCNWs, forming a loose SiCNW network without clogging [26]. The amount of deposited catalyst can be adjusted by controlling the AD reaction time. To achieve a better distribution of SiCNWs, a long catalyst evaporation time is necessary. In contrast, the SiCNWs catalysed by electroplated Ni particles can only grow on the surface of the preforms. All of the results demonstrate that the AD method is a novel and effective method for introducing highly uniform nanowires into the preform.

### 3.2. Densification of SiC_f_/SiC Composites

**Figure 7:**
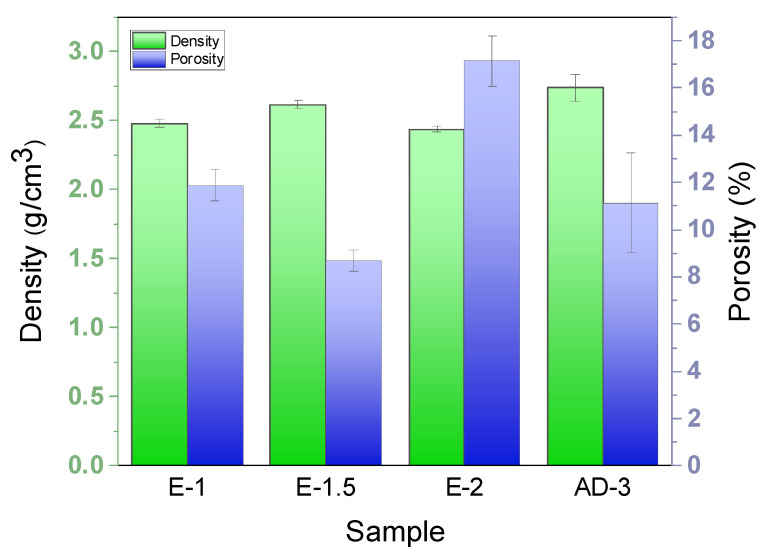
Histogram of the density and open porosity of the SiCNW-reinforced SiC_f_/SiC composites.

Figure 7 shows the density and open porosity of the SiCNW-reinforced SiC_f_/SiC composites prepared using different catalyst introduction methods. The E-1 composites with an EP current of 1 A had a density and open porosity of 2.48 g/cm^3^ and 11.9%, respectively. With an increase in current to 1.5 A, the E-1.5 composites showed a higher density of 2.62 g/cm^3^ and a lower porosity of 8.7%. However, with a further increase in current to 2 A, the E-2 composites showed a lower density of 2.44 g/cm^3^ and a higher porosity of 17.1%. Compared to the composites prepared using the EP catalyst addition process, the density of the AD-3 composite was significantly higher (2.74 g/cm^3^), with a moderate open porosity (11.1%).

**Figure 8:**
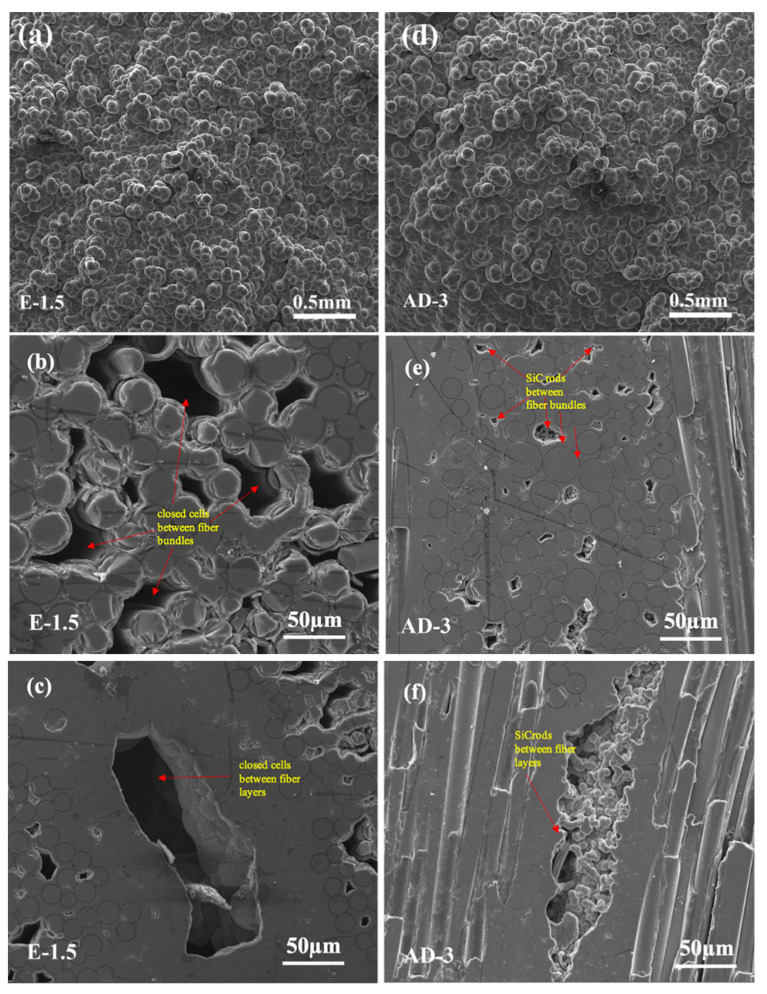
Surface and cross-sectional microstructures of SiCNW-containing SiC_f_/SiC composites after densification: (**a**) surface of E-1.5; (**b**) cross-section of inter-fiber bundles of E-1.5; (**c**) cross-section of fiber layer of E-1.5; (**d**) surface of AD-3; (**e**) cross-section of inter-fiber bundles of AD-3; and (**f**) cross-section of fiber layer of AD-3.

Figure 8 shows the microstructures of selected densified SiCNW-reinforced SiC_f_/SiC composites (E-1.5 and AD-3). Compared with the morphology obtained at 1200 °C [27], the morphology of SiC grains tends to be spherical or hexagonal at a 1050 °C deposition temperature (Figure 8a,d). With increasing density, the open pores of the composites are gradually blocked, forming closed cells, which hinder further densification and affect the mechanical properties of the composites (Figure 8b,c). Interestingly, SiC rods derived from SiCNWs and the SiC phase were observed in the internal pores of AD-3 (Figure 8e,f). The good infiltration ability of gaseous Ni atoms facilitated the homogeneous deposition of nanoscale Ni catalysts on the porous SiC_f_/SiC preform. As a result, SiCNWs were successfully introduced into the porous SiC_f_/SiC preform, forming a SiCNW network. The existence of the SiCNW network can increase the surface area and thus increase the effective deposition position of the SiC matrix, resulting in reduced porosity and enhanced density of the SiC_f_/SiC composites.

**Figure 9:**
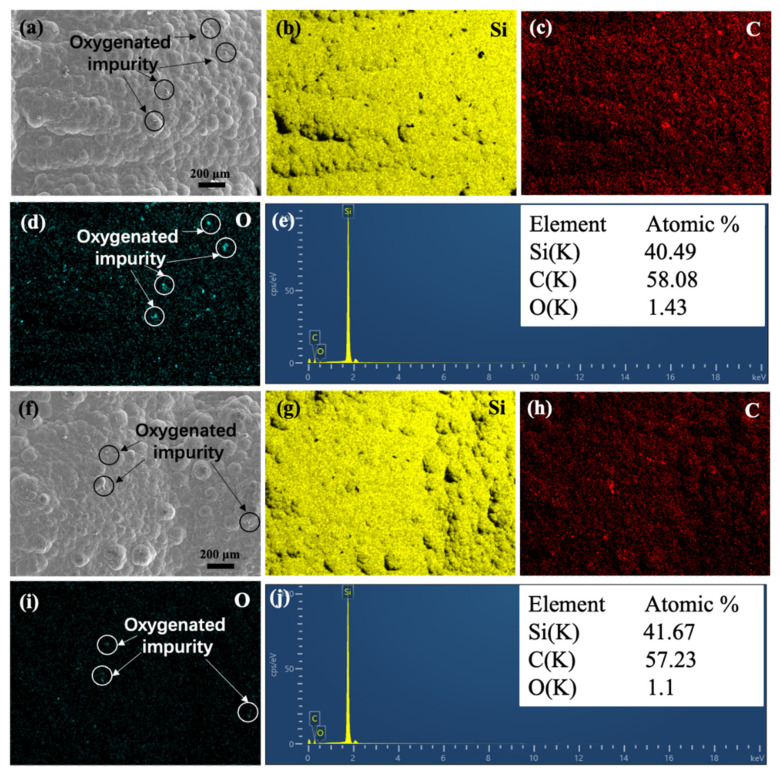
(**a**) sample E-2, (**b**–**d**) corresponding elemental mapping of Si, C and O, (**e**) corresponding EDX spectra and the atomic ratio of sample E-2, (**f**) sample AD-3, (**g**–**i**) corresponding elemental mapping of Si, C and O, (**j**) corresponding EDX spectra and the atomic ratio of sample AD-3.

The SiC matrix of samples E-2 and AD-3, shown in Figure 9a,f were selected for elemental mapping analysis, respectively. As shown in Figure 9b–d,g–i, the distribution of C and Si are uniformly distributed in the matrix of E-2 and AD-3. In addition, a small amount of O element was also existed due to the the adsorption of some oxygenated impurities. EDX analyses are shown in Figure 9e,j, reveals similar results that the atomic ratio of C and Si in the matrix of E-2 and AD-3 are close to 6:4.

### 3.3. Mechanical Properties of EP and AD SiC_f_/SiC Composites

**Figure 10:**
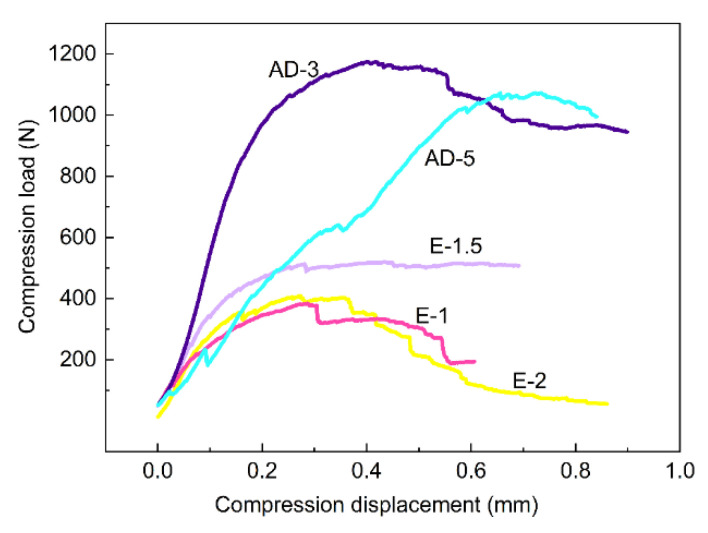
Load–displacement curves of SiCf/SiC composites containing SiCNWs.

Figure 10 shows the compressive load–displacement curves of SiCNW-reinforced SiC_f_/SiC composites prepared with the EP and AD methods. The load–displacement curves of the EP composites show that the maximum load and compressive displacement first increased and then decreased with increasing current, with maximum values of 516.2 N and 0.28 mm, respectively, for E-1.5. The EP composites were mainly reinforced by surface nanowires, and there were still many pores inside the composites. The ability of the composite to resist deformation was not improved since the SiCNW network was not successfully formed inside the EP composites. In contrast, the maximum load and compressive displacement greatly increased when a sufficient number of nanowires were formed using the AD method. When the deposition time of the SiCNWs was extended to 3 h, the maximum compressive load and displacement significantly increased, reaching 1175.0 N and 0.41 mm, respectively. When the deposition time was increased to 5 h, the maximum compressive load decreased slightly to 1070.0 N, while the compressive displacement increased to 0.73 mm. With increasing SiCNW growth time, the amount and aspect ratio of the SiCNWs increased (Figure 3c,d), and the SiCNW network was successfully formed inside the composites. These networks are cross-linked with each other to strengthen the matrix. The nanowires effectively inhibit the expansion of microcracks in the matrix, and the mechanical properties of the composites can be greatly improved. All of the results indicated that the inner SiCNW network has a significant effect on the mechanical properties of AD composites. In contrast, the strength and resistance to deformation of the AD composites at the appropriate nanowire growth time improved greatly, indicating that the AD method was more suitable for modifying the SiC_f_/SiC composites than EP.

All of the samples with Ni catalysts introduced by EP had similar pseudo-plastic fracture behaviour. Samples E-1.5 and AD-3 were taken as examples. The curves can be divided into three regions: (1) the initial elastic stage, (2) the nonlinear stage, and (3) the destructive damage stage. In the initial stage, the curves for both samples showed a similar trend. Under external load, the original micro-crack did not propagate, and the material underwent slight elastic deformation. In the nonlinear stage, crack initiation, propagation, deflection, and interface debonding occurred. As the crack propagated, energy-loss mechanisms, such as crack deflection, fiber bridging, SiCNW bridging, and debonding also occurred, which increased the toughness of the composite [28]. After the maximum load was reached, the composites entered the destructive stage. The curves for both E-1.5 and AD-3 showed a gradual decline, displaying typical pseudo-plastic fracture behaviour of the SiCNW-reinforced SiC_f_/SiC composites. As the external load continued to increase, the composite finally failed.

The compressive strengths of the SiC_f_/SiC composites, based on Equation (3), are summarised in Table 2. The compressive strengths of E-1, E-1.5, and E-2 demonstrate that the mechanical properties of the composites improved with increasing density. The compressive strength of AD-3 was the highest of all samples. Since the compressive strengths of samples AD-3 and AD-5 were similar, the corresponding compressive displacement of AD-5 increased by 78% and its load drop was negligible, indicating that the toughness of AD-5 had been improved [29,30].

**Figure 11:**
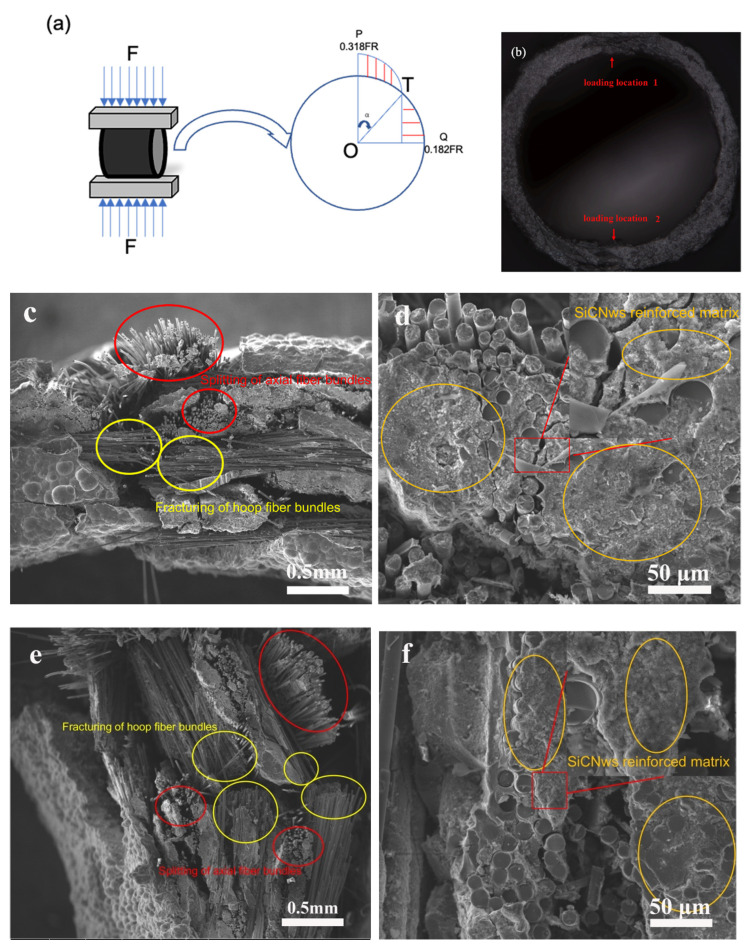
(**a**) Schematic of the bending moment distribution under radial compressive loading [31]; (**b**) fracture location of the SiC_f_/SiC composites; and (**c**–**f**) fracture morphology at (**c**,**d**) *α* = 0° and (**e**,**f**) *α* = 90°.

The compressive force and stress distribution of SiC clad tubes are schematically shown in Figure 11a. Detailed calculations and analyses using a finite element model (FEM) were presented in our previous paper [31]. According to the stress distribution at the loading position of the composites, the maximum circumferential compressive and tensile stresses are generated on the outer and inner surfaces of the sample, respectively. Moreover, under the action of circumferential stress, the loading position will fracture first. During the crushing test, the same fracture characteristics (Figure 11b) were observed as in the simulation results, indicating that the introduction of the SiCNWs did not alter the stress state of the annular specimens. The fracture morphologies at *α* = 0° and 180° are typical for the composite tube [32]. The fracture morphologies at different positions (*α* = 0° and 90°) are shown in Figure 11c–f. Under hoop stress, bending fracture occurred at *α* = 0° (Figure 11c). Under this stress state, both the hoop fibers and the matrix contribute to bearing the hoop tensile stress, which results in the fracture of the hoop fiber bundles. For the axial fiber bundles, the matrix bears most of the load, which results in the splitting of axial fiber bundles after the matrix breaks. However, as shown in Figure 11c,e, different locations showed a similar fracture pattern; only due to the different stress directions, the hoop fiber bundles collapsed towards the centre or expanded toward the outer surface at *α* = 0° and 90°, respectively. Many areas of the SiCNW-reinforced SiC matrix with cracks were observed at the fracture site (Figure 11d,f), indicating that when cracks appear in the matrix, the crack propagation energy was consumed due to the presence of SiCNWs. This proves that the SiCNWs played an important role in modification of the mechanical properties.

**Figure 12:**
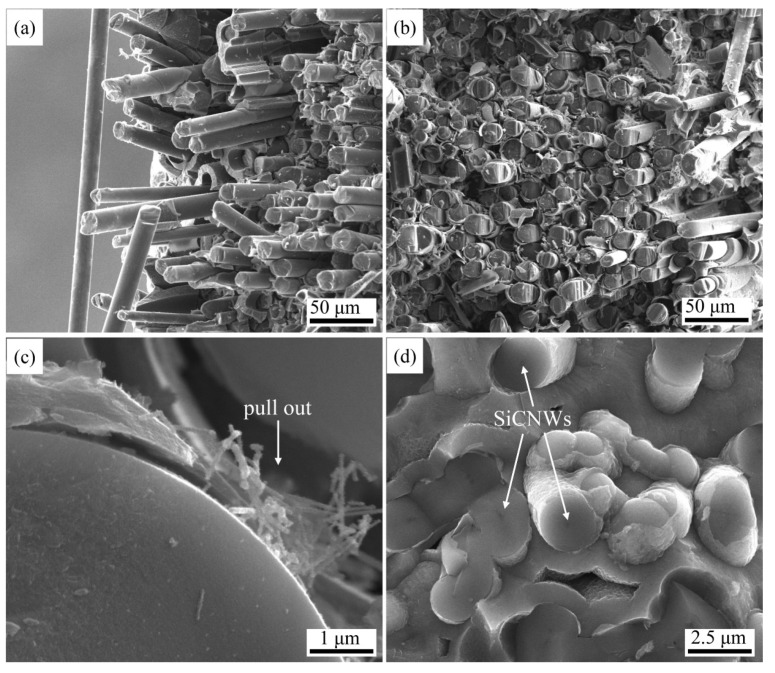
Fracture morphology of SiC_f_/SiC composites containing SiCNWs after compression tests: (**a**) E-1.5; (**b**) AD-3; (**c**,**d**) high-resolution images of AD-3.

Figure 12 shows the fracture morphologies of the compressed SiCNW-reinforced SiC_f_/SiC composites. In sample E-1.5, obvious fiber pull-out was observed, with an uneven fracture morphology (Figure 12a). For AD-3, the fracture was relatively uniform with few fibers pulled out (Figure 12b), indicating a high bonding strength between the fibers and matrix of the composite. The shear strength of the inner bundles decreased with increasing fiber pull-out length [33]. This shows that the nanowires introduced by AD more effectively increased the shear strength of the inner bundles than those introduced by EP. Since the riveting effect [26] between the SiCNWs and matrix increases the bonding strength between the fiber and matrix, the crack propagation energy could be effectively consumed by pull-out (Figure 12c) and fracture (Figure 12d) of the SiCNWs [11,34]. The fracture morphology of AD-3 (Figure 12d) indicates that many SiC rods were present and interlaced with each other to form a relatively dense matrix, filling the large internal pores. This can increase the density and enhance the toughness of the SiC_f_/SiC composite. The SiCNW toughening mechanism combined with the fiber-toughening mechanism can increase the toughness of the composites significantly.

## 4. Conclusions

In this paper, we presented an efficient catalyst introduction method for uniformly growing SiCNWs in SiC_f_ preforms assisted by the AD of a Ni catalyst. Compared with the EP-composites and SiC_f_/SiC composites, the compressive strength of the AD-composites is increased by 51.1% and 56.0%, respectively. Owing to the notable diffusivity of Ni atoms during AD, a SiCNW network formed among the internal fiber bundles and between the SiC fiber layers with an appropriate reaction time (3–5 h). The increased strength and toughness were attributed to the riveting effect between the SiCNWs and matrix, which can effectively consume energy during crack propagation. These findings suggest that the AD method based on Ni-diffusion successfully ameliorates the shortcomings of EP. The AD method provides several advantages for introducing metal catalysts into SiC_f_ preforms for various applications and increases the mechanical properties of SiC_f_/SiC composites.

## Figures and Tables

**Table 1:** Parameters for the in-situ growth of SiCNWs and CVI process.

	Sample	E-1	E-1.5	E-2	AD-1	AD-3	AD-5
Process							
Electroplating process	Electroplating substrate	SiCf preform			Graphite sheet		
	Bath composition	initial concentration			1/16 of initial concentration		
	Current	1 A	1.5 A	2 A	1 A		
	Time	2 min			2 min		
SiCNW growthprocess	Temperature	1000–1100 °C			1000–1100 °C		
	Pressure	2000 Pa			2000 Pa		
	Carrier gas	50 sccm			50 sccm		
	Dilution gas	750 sccm			750 sccm		
	Reaction time	2 h			1 h	3 h	5 h
CVI densification process	Temperature	1050 °C			1050 °C		
	Pressure	400–600 Pa			400–600 Pa		
	Carrier gas H2	450 sccm			450 sccm		
	Carrier gas Ar	300 sccm			300 sccm		
	Time	100 h			100 h		

**Table 2:** Mechanical properties of SiC_f_/SiC composites prepared by EP and AD methods.

Sample	Compressive Load (N)	Compressive Displacement (mm)	Compressive Strength (MPa)
SiC_f_/SiC after 150 h densification	429.15	0.39	225.94 (±5.49)
E-1	384.1	0.29	181.27 (±12.43)
E-1.5	516.2	0.28	228.66 (±9.85)
E-2	408.5	0.27	125.01 (±6.63)
AD-3	1175.0	0.41	352.36 (±10.28)
AD-5	1070.0	0.73	345.41 (±7.26)

## Data Availability

The data used to support the findings of this study are available from the paper.
